# Identification of tannic cell walls at the outer surface of the endosperm upon Arabidopsis seed coat rupture

**DOI:** 10.1111/tpj.14994

**Published:** 2020-10-15

**Authors:** Lara Demonsais, Anne Utz‐Pugin, Sylvain Loubéry, Luis Lopez‐Molina

**Affiliations:** ^1^ Department of Botany and Plant Biology University of Geneva Geneva Switzerland; ^2^ Institute of Genetics and Genomics in Geneva (iGE3) University of Geneva Geneva Switzerland

**Keywords:** *Arabidopsis thaliana*, seed, tannic cell wall, apoplastic barrier, seed coat, endosperm

## Abstract

The seed coat is specialized dead tissue protecting the plant embryo from mechanical and oxidative damage. Tannins, a type of flavonoids, are antioxidants known to accumulate in the Arabidopsis seed coat and *transparent testa* mutant seeds, deficient in flavonoid synthesis, exhibit low viability. However, their precise contribution to seed coat architecture and biophysics remains evasive. A seed coat cuticle, covering the endosperm outer surface and arising from the seed coat inner integument 1 cell layer was, intriguingly, previously shown to be more permeable in *transparent testa* mutants deficient not in cuticular component synthesis, but rather in flavonoid synthesis. Investigating the role of flavonoids in cuticle permeability led us to identify periclinal inner integument 1 tannic cell walls being attached, together with the cuticle, to the endosperm surface upon seed coat rupture. Hence, inner integument 1 tannic cell walls and the cuticle form two fused layers present at the surface of the exposed endosperm upon seed coat rupture, regulating its permeability. Their potential physiological role during seed germination is discussed.

## Significance Statement

Tannins are seed coat antioxidants promoting seed viability and dormancy. A cuticle, covering the endosperm outer surface was previously shown to be more permeable in *transparent testa* mutants, which are deficient in flavonoid synthesis but not in cuticular component synthesis. We show that the endosperm outer surface is also covered by tannic cell walls arising from the seed coat inner integument 1 layer. Thus, tannic cell walls are endosperm outer surface structural components influencing its permeability.

## INTRODUCTION

Seeds are a late land plant evolution innovation consisting of plant embryos maintained in a desiccated and highly sheltered state surrounded by a protective dead seed coat of maternal origin. Embryo survival only relies on the pre‐established highly resistant mature seed state that notably depends on the protective seed coat. Although seeds are desiccated and metabolically inert structures, their exposure to atmospheric oxidation will irremediably lead to oxidative damage. Besides the mechanical protective function, the seed coat fulfills the need to shield living tissues from excessive exposure to atmospheric oxygen and is obligatory to preserve the long‐term capacity of the seed to produce a viable seedling. Furthermore, the seed coat also regulates the entry of water upon seed imbibition, potentially to discriminate partial or short‐term water exposure from more prolonged imbibition (Windsor *et al*., 2000; De Giorgi *et al*., 2015). Hence, understanding the biophysical and anatomical properties of the boundary demarcating the seed’s living tissues from the environment is essential to understand seed physiology and more generally plant fitness.

**Figure 1:**
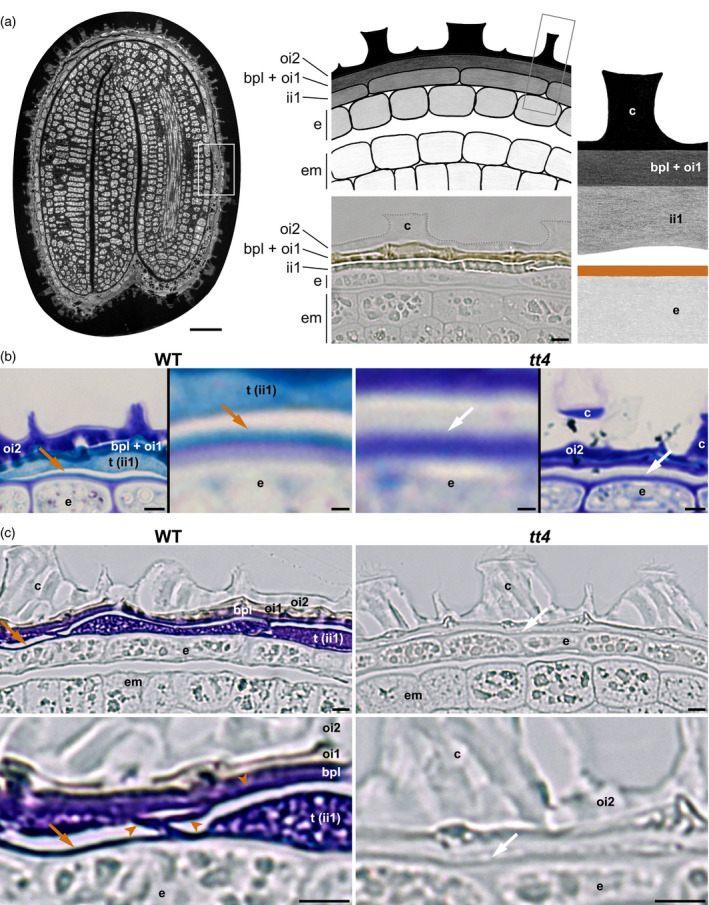
Presence of tannins at the outer surface of the endosperm. (a) Left, semi‐thin section of a wild‐type (WT) seed (autofluorescence). Center top, schematic drawing illustrating the organization of the seed peripheral area (corresponding to the white‐boxed region in the left panel). Center bottom, micrograph of an unstained semi‐thin section showing the seed peripheral area; ii1, the bpl, oi1 and oi2 form the seed coat. Right panel, schematic drawing of an enlarged seed coat area (gray‐boxed region in the center top panel), the brown line indicates the localization of tannins along the endosperm outer surface (arrows in b,c, and in Figure S1a,c). Drawings were not made to scale to visualize the positions of the different cells and structures of interest better. (b) Semi‐thin sections of WT seeds (left panels) and *transparent testa (tt)4* seeds (right panels) stained with toluidine blue; in each case, a global view of the seed coat and an enlargement of the region around the arrow is shown. (c) Semi‐thin sections of WT seeds (left panels) and *tt4* seeds (right panels) stained with 4‐dimethylaminocinnamaldehyde; in each case, a global view of the seed coat and an enlargement of the region around the arrow is shown below. Brown arrowheads in bottom left panel indicate the reticulated structure formed by ii1 tannic cell walls. In (b) and (c), brown arrows (left panels) indicate the detection of tannins along the endosperm outer cell wall; white arrows (right panels) indicate no detection of tannins along this cell wall. Scale bars = (a) 50 μm (left) and 5 μm (center bottom); (b) 5 and 1 μm for general views and magnified insets, respectively; (c) 5 μm. bpl, brown pigment layer; c, columella; e, endosperm; em, embryo; ii1, inner integument 1; oi1, outer integument 1; oi2, outer integument 2; t, tannin blocks.

In *Arabidopsis thaliana*, the mature seed consists of an external dead seed coat surrounding a single cell layer of endosperm itself surrounding the embryo. The seed coat arises from the differentiation of ovular integuments after double fertilization that produces the endosperm and zygote. Seed germination takes place upon seed imbibition and is defined by concomitant embryonic radicle emergence and endosperm rupture. However, seed coat rupture is the first visible event before seed germination and therefore defines the time whereupon the endosperm is exposed to the outer environment. During ovular differentiation accompanying seed development, three cell layers of inner integuments (ii1, ii1′ and ii2) and two cell layers of outer integuments (oi1 and oi2) undergo complex modifications, giving rise to the mature seed coat (Figure 1a). Inner and outer ovular integuments will contribute distinct key components of the mature seed coat such as the production of tannins and mucilage, respectively. The seed coat brown pigment layer (bpl) arises from the progressive collapse of ii1′ and ii2 cells during late seed development (Figure 1a) (Beeckman *et al*., 2000; Debeaujon *et al*., 2007).

At early seed developmental stages, ii1 cells, differentiating alongside the developing endosperm, begin to accumulate proanthocyanidins, a type of oligomeric flavonoids also known as condensed tannins, and hereafter referred to as tannins. After accumulating in vacuoles during early seed development, tannins impregnate cell walls of not only ii1 cells but also those of the bpl in the course of seed coat differentiation (Beeckman *et al*., 2000; Kitamura *et al*., 2004; Pourcel *et al*., 2007).

Debeaujon *et al*. have demonstrated the importance of tannins in seed coat physiology. Indeed, a suite of *transparent testa* (*tt*) mutants, deficient in flavonoid synthesis and producing colorless seeds, have low dormancy and viability as well as premature seed coat rupture (Debeaujon *et al*., 2000). *tt* mutants exhibit enhanced permeability to the dye tetrazolium red and, accordingly, these defects can be associated with increased oxidative damage (Landry *et al*., 1995; Debeaujon *et al*., 2000; MacGregor *et al*., 2015). Importantly, *tt* mutant phenotypes are maternally inherited and therefore the phenotypes are due to a deficient seed coat. A number of explanations have been put forward regarding the function of flavonoids in seeds. Flavonoids act as antioxidants and therefore their absence could explain the increased oxidative damage observed in *tt* mutant seeds. Furthermore, it was hypothesized that during seed desiccation, oxidation of phenolic compounds, such as tannins, would reduce seed coat permeability (Marbach and Mayer, 1974; Stafford, 1974; Marbach and Mayer, 1975; Werker *et al*., 1979).

Cuticles are fatty and waxy hydrophobic films covering the plant surface (Yeats and Rose, 2013). Recently, a cuticle tightly associated with the outer cell wall of the endosperm in mature seeds was identified (De Giorgi *et al*., 2015). Remarkably, this cuticle is maternal, being produced by ii1 cells during seed development and transferred to the endosperm around the heart and torpedo stages of embryo development (Loubéry *et al*., 2018). Genetic studies showed that mutants defective in cuticle biosynthesis have low viability and dormancy levels, which correlates with increased oxidative stress in mature seeds (De Giorgi *et al*., 2015). In addition, similar to *tt* mutants, cuticle biosynthesis mutants prematurely ruptured their seed coat upon seed imbibition because of abnormal endosperm cell expansion (De Giorgi *et al*., 2015). Furthermore, in seed coat‐ruptured seeds, the endosperm of cuticle biosynthesis mutants was shown to be more permeable to toluidine blue, a dye commonly used to assess cuticle integrity (Tanaka *et al*., 2004; De Giorgi *et al*., 2015). These observations are consistent with the notion that a deficient endosperm‐associated cuticle disrupts the seed’s water uptake dynamics and increases the seed’s permeability to oxygen.

However, a number of unsolved questions regarding the endosperm‐associated cuticle remain unaddressed. First, whether the maternal cuticle continues to be associated with the endosperm upon seed imbibition and therefore, whether it directly contributes to its permeability was not investigated. Secondly, Loubéry *et al*. reported that the endosperm of *tt* mutant seeds also showed increased toluidine blue permeability, suggesting that the endosperm‐associated cuticle is deficient in *tt* mutants (Loubéry *et al*., 2018). How a deficiency in synthesis of flavonoids impacts the permeability of the endosperm‐associated cuticle was not investigated. Histological experiments suggested that tannins were present on the outer surface of the endosperm (Loubéry *et al*., 2018). However, whether their presence simply reflects non‐specific binding to the endosperm outer surface following their release after seed coat rupture was not investigated. Alternatively, whether tannins are bona fide components of the endosperm‐associated cuticle is unknown. Hence, whether tannins directly or indirectly participate in the permeability of the cuticle remains to be understood. Addressing this specific question would provide insights about how tannins generally contribute to the seed coat biophysical properties.

Here we show that the endosperm‐associated cuticle is part of a previously uncharacterized seed structure. The outer surface of the endosperm is covered with a bilayer of electron‐lucent and electron‐dense material (De Giorgi *et al*., 2015). We show that the former corresponds to the cuticle, whereas the latter is a cell wall impregnated with tannins, which originates from ii1 cells and is referred to here as the tannic cell wall. We show that tannic cell walls remain attached to the endosperm upon seed coat rupture, thus participating in its permeability properties. Furthermore, tannic cell walls leave a characteristic imprint of ii1 cell wall material on the outer endosperm surface; this reveals that during seed coat rupture, the ii1 cell walls anticlinal to the endosperm surface are breaking points between the inner seed tissues and the seed coat. Collectively, our findings shed new light on how the biophysical properties of the Arabidopsis seed coat are implemented by means of a cuticle and tannic cell walls.

## RESULTS

It was shown using *tt* mutant seeds, deficient in flavonoid synthesis, that flavonoids are necessary to confer normal impermeability to the outer surface of the endosperm and, by extension, to the endosperm‐associated cuticle (Loubéry *et al*., 2018). However, whether the involvement of flavonoids is direct, i.e. as intrinsic components of the cuticle, or indirect was not determined. Furthermore, whether the endosperm‐associated cuticle remains attached at the surface of the endosperm upon rupture of the seed coat was not ascertained. Here we sought to address these two questions in turn.

### Presence of tannins at the outer surface of the endosperm

Histological sections by Loubéry *et al*. suggested that the surface of mature endosperm cells contains tannins, as it appeared brown in wild‐type (WT) seeds but not in *tt4* mutants, lacking the enzyme for the first step of flavonoid biosynthesis and hence lacking tannins (Feinbaum and Ausubel, 1988; Loubéry *et al*., 2018). However, the resolution of the histological preparations did not allow identifying unambiguously the subcellular structure harboring this color, i.e. to discriminate whether it was the cuticle itself that was brown or another closely apposed structure. To explore these possibilities further, we sought to clarify the putative presence of tannins along the endosperm surface (brown line in Figure 1a, right), using a combination of genetics and improved histological methods.

We used higher resolution semi‐thin sections of Epon‐embedded mature dry seeds stained with toluidine blue and methylene blue, two dyes that are known to recognize tannins (Okuda *et al*., 1985; Debeaujon *et al*., 2003; Nesi *et al*., 2009; Sánchez‐Martín *et al*., 2010; Qu *et al*., 2013; Wang *et al*., 2019). These metachromatic dyes revealed tannins in the ii1 cell layer with blue (toluidine blue) and turquoise (methylene blue) colors (Figures 1b and S1a, left panels). Strikingly, they also revealed a linear structure along the outer surface of the endosperm that shared the same color as that of tannins (Figures 1b and S1a, left panels). The similarity in color obtained with two different metachromatic dyes suggests a similarity in composition; thus, these data suggest that the linear structure might contain tannins. Consistently, these linear structures were not colored in sections of *tt4* mature seeds, lacking tannins, upon staining with the same dyes (Figures 1b and S1a, right panels). Similarly, in unstained WT seed sections, a dark beige line was clearly visible along the endosperm surface that had the same color and shade as that of the tannins present in the ii1 cell layer (Figure S1b, left panels). By changing the focal plane, the line showed a yellowish color, which was similar to that shown by the tannins (Figure S1c, left panels), and as above, it was not visible in *tt4* mutants (Figure S1b,c).

4‐dimethylaminocinnamaldehyde (DMACA) is a dye that binds tannins and its late precursors (flavan‐3‐ols) with high specificity, as it does not bind other flavonoids such as flavonols or anthocyanidins (Cadot *et al*., 2006; Auger *et al*., 2010; Hammouda *et al*., 2014). DMACA presents a significantly better sensitivity than vanillin, another dye commonly employed to detect tannins (Li *et al*., 1996); besides, its purple/deep blue signal is best‐suited for Arabidopsis seed coat histological studies, compared with vanillin whose light orange/pink signal can easily be confused with the natural brownish color of the seed coat (Li *et al*., 1996; Abrahams *et al*., 2002; Kitamura *et al*., 2004). Upon DMACA staining of WT semi‐thin sections, tannins in ii1 cells gave a purple signal; in addition, a purple signal was visible on a linear structure along the outer surface of the endosperm (Figure 1c, left panels). As above, this linear signal was not visible in *tt4* mutant semi‐thin sections (Figure 1c, right panels).

Overall, these results demonstrate the presence of tannins at the outer surface of endosperm cells in Arabidopsis mature seeds. Next, we sought to identify which structures at the outer surface of the endosperm contain tannins.

### ii1 cell walls are tannified and form a continuous reticulated tannic apoplastic barrier tightly bound to the endosperm‐associated cuticle

In previous reports, transmission electron microscopy (TEM) images revealed the presence of a two‐layered structure covering the outer surface of mature endosperm cells, consisting of an inner electron‐lucent layer and an outer electron‐dense layer (De Giorgi *et al*., 2015; Loubéry *et al*., 2018). The two layers were interpreted as representing different compositions of the endosperm‐associated cuticle. Furthermore, the electron‐dense layer periodically protruded outwardly, forming an extended electron‐dense reticulated structure surrounding the endosperm (purple dashed line in Figure S2). Loubéry *et al*. proposed that this reticulated structure is an apoplastic network consisting of electron‐dense cuticular material enclosing ii1 cells. However, Loubéry *et al*. noted that this is inconsistent with the fact that this structure was not recognized by the cuticle dye Auramine O (Loubéry *et al*., 2018). The nature and the composition of these layers and that of the reticulated structure were not investigated further.

**Figure 2:**
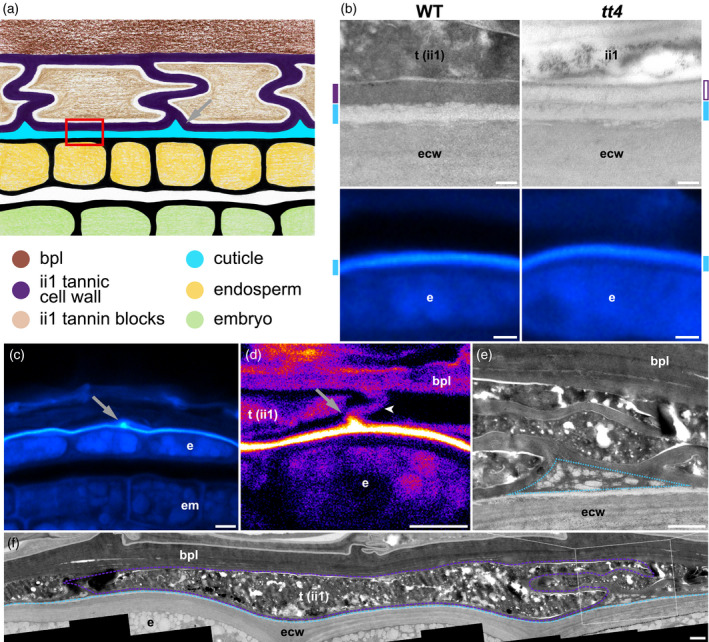
ii1 cell walls are tannified and form a continuous reticulated tannic apoplastic barrier tightly bound to the endosperm‐associated cuticle. (a) Schematic drawing illustrating the organization of the endosperm outer surface area. The drawing is not made to scale to visualize the positions of the different structures of interest better. The red‐boxed region indicates the surface of the endosperm where the cuticle and inner tannified cell wall are tightly associated. The gray arrow indicates the location of cuticular accumulation. (b) Transmission electron microscopy (TEM) micrographs of the endosperm outer surface region (top row) and paraffin sections stained with Auramine O (bottom row) in wild‐type (WT) and *tt4* mutants, corresponding to the red‐boxed region shown in (a). Cyan marks indicate the position of the cuticle and purple marks indicate the position of the ii1 inner periclinal cell wall (filled in purple and in white when it is electron‐dense and electron‐lucent, respectively). (c) Auramine O staining of a WT seed paraffin section; the arrow indicates a local signal accumulation corresponding to the region indicated by the gray arrow in (a). (d) Confocal micrograph of a WT seed paraffin section stained with Auramine O. The arrow indicates a local signal accumulation corresponding to the region indicated by the gray arrow in (a); the arrowhead indicates an anticlinal cell wall between two adjacent ii1 cells. (e) TEM micrograph of a junction between ii1 cells in a WT seed (enlargement of the white‐boxed region in f). The dashed blue triangle indicates the region where droplet‐like electron‐lucent material accumulates, between neighboring ii1 cells and the cuticle. (f) TEM micrograph presenting a global view of the WT seed inner integuments. The purple dashed line indicates the cell wall of one ii1 cell (electron‐dense material) and the light blue dashed line indicates the cuticle (electron‐lucent material). The original picture is in Figure S5a. Scale bars = (b) 200 nm and 2 μm, top and bottom rows, respectively; (c,d) 5 μm; (e,f) 1 μm. bpl, brown pigment layer; e, endosperm; ecw, endosperm cell wall; em, embryo; ii1, inner integument 1; t, tannin blocks.

Here, we challenge the notion that the electron‐dense and electron‐lucent layers represent different compositions of a cuticle. Rather, we propose that only the electron‐lucent layer is a bona fide cuticle; we also propose that the electron‐dense layer corresponds to ii1 cell walls that have undergone tannin impregnation (Figure 2a). Hereafter, we refer to tannin‐impregnated cell walls as tannic cell walls. Therefore, this model states that during seed development, the ii1 cells produce a cuticle on their inner side, while their primary cell walls become impregnated with tannins. Eventually, the inner cuticle and inner tannified cell wall become tightly associated with the outer surface of the endosperm (red‐boxed region in Figure 2a). The remaining ii1 tannified walls form the reticulated structure described by Loubéry *et al*., which generates a continuous tannic barrier (Figures 2a and S2). In turn, in each ii1 cell, the tannic cell walls enclose the tannic depositions that are visible as rugged electron‐dense blocks in TEM.

To evaluate this model, we first assessed whether the electron‐dense reticulated structure corresponds to tannified cell walls. Interestingly, the linear structure revealed by DMACA at the surface of the endosperm periodically protruded outwardly, forming a reticulated extended structure in a manner most similar to that observed with the electron‐dense layer in TEM (arrowheads in Figure 1c left; Figure S3a). Furthermore, DMACA did not reveal a reticulated structure in *tt4* mutant seed sections (Figure 1c, right panels). In addition, the electron‐dense reticulated structure was no longer electron‐dense in *tt4* mutants (Figure 2b, top row). These observations strongly indicate that the reticulated structure revealed by DMACA corresponds to the electron‐dense structure observed in TEM.

Calcofluor white (CW) is a fluorescent dye used for staining plant cell walls due to its specific binding to cellulose (Hughes and McCully, 1975). Semi‐thin WT seed sections stained with CW revealed the presence of linear plant cell wall material periodically protruding outwardly from the surface of the endosperm; this is consistent with the notion that it corresponds to ii1 cell wall material (arrowheads in Figure S4). Furthermore, the CW signal formed a reticulated structure reminiscent of the one observed with DMACA and TEM (Figure S4). Overall, these observations show that the electron‐dense reticulated structure corresponds to tannified cell walls.

We next assessed whether the electron‐lucent layer corresponds to the endosperm‐associated cuticle. We observed that the electron‐lucent layer had the same appearance in both WT and *tt4* mutants, consistent with previous reports (Figure 2b, top row) (Loubéry *et al*., 2018). Similarly, Auramine O, a fluorescent cuticle marker (Lequeu *et al*., 2003; Szczuka and Szczuka, 2003; Buda *et al*., 2009), revealed the presence of a linear signal that covers the outer surface of the endosperm; furthermore, it had the same appearance in both WT and *tt4* seeds (Figure 2b, bottom row). Thus, unlike the electron‐dense/DMACA structure, the appearance of the electron‐lucent/Auramine O layer is not perturbed by the absence of tannins, indicating that they correspond to distinct structures. Furthermore, these results strongly support the notion that the electron‐lucent layer and the Auramine O signal represent the same structure, namely the endosperm‐associated cuticle.

To ascertain this claim further, we took advantage of an unexpected observation. Indeed, we observed that the Auramine O linear signal was occasionally punctuated by patches protruding outwardly (Figure 2c). Confocal micrographs suggested that these patches are present at the meeting location of two tannic cell walls from adjacent ii1 cells (Figure 2d). If the electron‐lucent material corresponds to the Auramine O structure, then the existence of Auramine O patches predicts that corresponding electron‐lucent depositions should be visible in TEM and that they should be located at the meeting point of adjacent ii1 cells.

Accordingly, TEM images confirmed that at the meeting point of two adjacent ii1 cells, electron‐lucent material accumulated on the outer side of the cuticle (Figure 2e,f). Indeed, after protruding out from the surface of the endosperm and before fusing, two tannic cell walls from adjacent ii1 cells form two edges of a triangle whose third edge is the electron‐lucent layer that remains associated with the surface of the endosperm (Figure 2e,f). Electron‐lucent material accumulated in the interior of the triangle, taking the form of an assembly of droplet‐like structures (Figure 2e). We noticed that the number and surface of these droplet‐like structures varied considerably among the various triangular locations, and they were sometimes absent (Figure S5b). This is consistent with the fact that the patches of signal revealed by Auramine O were also not systematically visible along the outer surface of the endosperm. These observations provide further correlative evidence that the electron‐lucent layer corresponds to the endosperm‐associated cuticle.

Altogether, we conclude that the outer endosperm surface is covered by a two‐layered structure consisting of a bona fide cuticle and a tannic cell wall, both derived from ii1 cells. ii1 cell walls are tannified and further protrude outwardly, forming a continuous tannic reticulated apoplastic barrier surrounding the seed’s living tissues.

### The ii1 inner periclinal tannic cell wall remains attached to the endosperm after seed coat rupture

We previously showed that the outer surface of the endosperm is more permeable to toluidine blue in both *bodyguard* mutants, deficient in cuticle formation, and *tt4* mutants (De Giorgi *et al*., 2015; Loubéry *et al*., 2018). Together with the model proposed above, these observations would therefore support the notion that both the cuticle and the tannic cell wall contribute to the biophysical properties of the outer surface of the endosperm. However, to ascertain this claim, we sought to verify that both maternal structures remain present at the outer surface of the endosperm upon seed coat rupture, which was not previously investigated.

**Figure 3:**
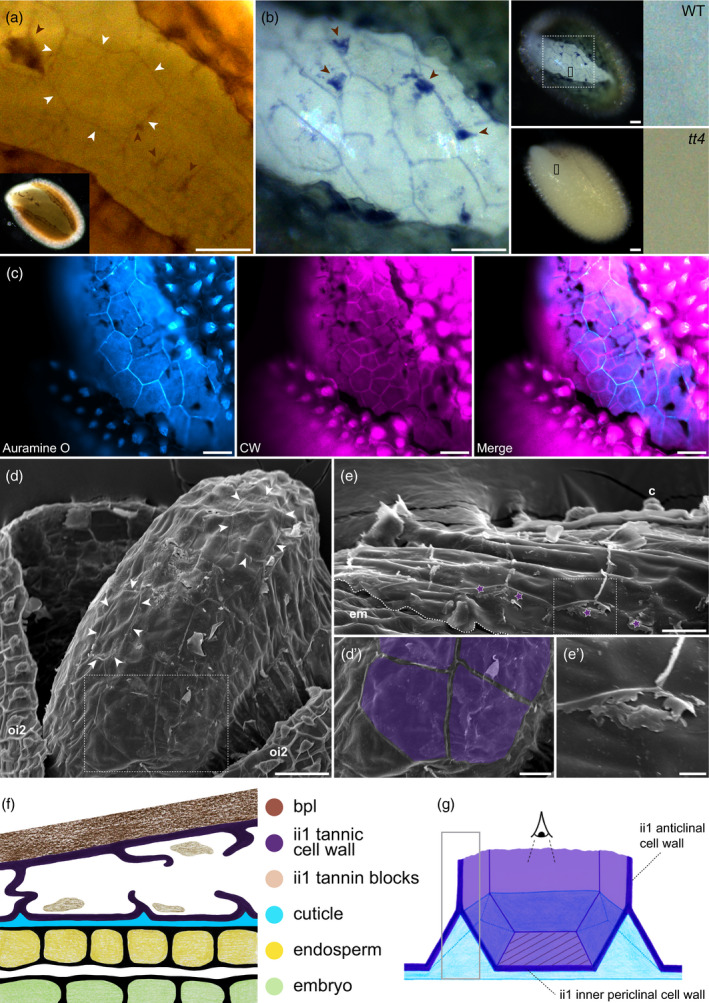
The ii1 inner periclinal tannic cell wall remains attached to the endosperm after seed coat rupture. (a) Unstained seed coat‐ruptured wild‐type (WT) seed showing a polygonal pattern present at the outer surface of the endosperm. White arrowheads delineate a particular polygonal contour. (b) 4‐Dimethylaminocinnamaldehyde staining of seed coat‐ruptured WT and *tt4* seeds. The left panel is an enlargement of the dashed‐boxed area shown in the WT seed picture of the middle panel. The right panels are enlargements of the small black boxes shown in the middle panel. In (a,b) brown arrowheads indicate remnants of ii1 tannin blocks (see Discussion). (c) Seed coat‐ruptured WT seed co‐stained with Auramine O and CW. (d,e) Scanning electron microscopy micrographs of seed coat‐ruptured (d) and endosperm‐ruptured (e) WT seeds. White arrowheads indicate the visible remnants of ii1 cell walls. (d′) Enlargement of the boxed region in (d), where ii1 inner periclinal wall surfaces were colorized in purple. (e′) Enlargement of the boxed region in (e), where remnants of ruptured ii1 anticlinal cell walls are visible (indicated by purple stars). Note that scanning electron microscopy was performed on fresh (non‐fixed) material; because of the microscope vacuum, ruptured seed coats would close themselves, hiding zones of interest (see Figure S8b for an example); to remedy this, we had to increase the aperture of seed coat‐ruptured seeds manually, thus uncovering a wider space than that corresponding to a normal seed coat‐ruptured seed. (f) Schematic drawing of seed coat organization during seed coat rupture (outer integuments are not represented). (g) Schematic drawing in 3D of one ii1 cell during seed coat rupture. The gray‐boxed region corresponds to the signal given by DMACA (b) and Auramine O (c) along broken anticlinal ii1 walls. The hatched area indicates the ii1 inner periclinal cell wall surface, which becomes the first barrier facing the environment during seed coat rupture. Drawings (f,g) were not made to scale to better visualize the structures of interest. Scale bars = (a–d), 50 μm; (d′,e) 20 μm; (e′) 5 μm. c, columella; em, embryo; ii1, inner integument 1; oi2, outer integument 2

In unstained WT seeds undergoing seed coat rupture, the exposed outer endosperm surface was covered by an unanticipated and striking pattern of brown‐edged polygons, which was not previously reported (Figure 3a). When WT seeds were stained with DMACA the polygonal pattern was even more striking, as DMACA strongly colored the polygon edges (Figure 3b, left panel). Furthermore, the interior of the polygons was also lightly stained by DMACA and this coloration was not visible in *tt4* mutants (Figure 3b, right panels). These observations provided a first indication that the surface of the endosperm remains entirely covered by ii1 inner periclinal tannic cell walls during seed coat rupture, notably as manifested by the tannin‐specific DMACA coloration of the polygon interiors.

The presence of a pattern of brown‐edged polygons at the outer surface of endosperm cells, further exacerbated by DMACA, strongly suggested that anticlinal ii1 tannic cell wall material remains attached to the ii1 inner periclinal tannic cell wall. Hence, the polygonal pattern would result from additional ii1 anticlinal cell wall material, which in turn would generate an imprint of the ii1 cell contours on the outer surface of the endosperm (Figure 3a,b). Incidentally, this suggestion would imply that the anticlinal ii1 tannic cell walls are the rupture points during seed coat rupture, which was not previously reported. We sought to evaluate these claims further by monitoring cuticular material at the surface of the endosperm. Indeed, we saw above that patches of cuticular accumulation are found on the outer side of the cuticle where two adjacent ii1 cells meet (Figure 2c,e). Therefore, the model predicts that this cuticular accumulation should also generate a polygonal pattern at the outer surface of endosperm cells corresponding to an imprint of ii1 cells.

Seeds that had undergone seed coat rupture were stained with the cuticle dye Auramine O. As anticipated by the model, this also revealed a striking polygonal pattern at the outer surface of the endosperm similar to that seen with DMACA (Figure 3c, left panel). Demonstrating that the Auramine O polygonal pattern matches a polygonal pattern generated by the ii1 cell wall would require co‐staining whole‐mount seed coat‐ruptured seeds with Auramine O and CW to show that the polygonal edges coincide. However, as shown in Figure S4, ii1 cell walls are very weakly stained with CW compared with endosperm cell walls. In addition, the very small distance between the endosperm outer cell wall and the ii1 inner periclinal cell wall is below the resolution of the light microscope. Therefore, it is expected that in whole‐mount stainings, the signal given by CW on ii1 inner periclinal cell walls will be hidden by the much stronger signal given by the endosperm outer cell wall. Indeed, CW enabled visualizing endosperm cell walls and, deeper within the seed, embryo cell walls, but it did not reveal ii1 cell walls (Figure 3c, middle panel; Figure S6).

Therefore, we opted for an indirect approach. The polygonal pattern revealed by Auramine O at the outer surface of the endosperm corresponds to either the anticlinal endosperm cell walls or the anticlinal ii1 cell walls. Thus, if the polygonal pattern revealed by Auramine O corresponded to endosperm anticlinal walls, it would co‐localize with the signal given by these walls upon CW staining. Strikingly, the Auramine O polygonal pattern was seen in close vicinity to endosperm cell walls and slightly above them (Figure 3c). Furthermore, their respective patterns did not match, demonstrating that the Auramine O signal does not delineate endosperm cells (Figure 3c, right panel). This is further evidenced by the fact that endosperm cells and ii1 cells have a substantial size difference: ii1 cells are significantly larger than endosperm cells (Figures 1c, 2f and S1–S6). This size difference was observed between the contours delineated by DMACA and Auramine O, and those of endosperm cells revealed by CW (Figure 3b,c). Hence, we conclude that the polygonal pattern revealed by Auramine O corresponds to an imprint of ii1 cells at the outer surface of the endosperm.

Overall, these data are strongly consistent with the notion that the endosperm‐associated cuticle and the inner periclinal tannic ii1 cell walls remain attached to the endosperm surface upon seed coat rupture.

To corroborate this claim further, we used scanning electron microscopy (SEM) on seed coat‐ruptured seeds. Again, a polygonal pattern could be recognized at the surface of the endosperm (Figures 3d,d′ and S7). The polygons matched in size and shape those described above without dye or with DMACA and Auramine O (Figure 3a,c). Furthermore, in some regions of the outer endosperm surface, underlying endosperm cells collapsed partially, probably because of the vacuum of the SEM, which generated a hollow pattern that revealed the endosperm cell contours. The endosperm cell contours were significantly smaller than the polygons observed at their surface (Figure S8a). These observations are consistent with the model described above, namely that ii1 anticlinal cell walls remain attached at the outer surface of the endosperm leaving an imprint of ii1 cell contours.

This conclusion is further supported by the following observation: close inspection revealed that the polygonal edges observed in SEM had sheet‐like appearance and quality (Figure 3e,e′). The extension of this sheet‐like material varied along the polygonal contours. These observations support the notion that the sheet‐like material corresponds to ruptured ii1 anticlinal cell walls, which consequently leaves portions of cell walls of different sizes resting on the ii1 inner periclinal cell walls that cover the endosperm‐associated cuticle (Figure 3f).

Overall, these observations corroborate the notion that during seed coat rupture, the endosperm‐associated cuticle and the ii1 inner periclinal tannic cell wall remain attached to the outer surface of the endosperm after ii1 anticlinal cell wall rupture (Figure 3f). The juxtaposed cuticle and ii1 inner periclinal tannic cell wall two‐layer structure constitutes the first physical barrier separating living tissues from the outer environment (Figure 3f,g). Together with the toluidine blue permeability experiments reported by De Giorgi *et al*. and Loubéry *et al*. (De Giorgi *et al*., 2015; Loubéry *et al*., 2018), these observations show that both the cuticle and the tannic cell wall of this two‐layer structure contribute to the biophysical properties of the outer surface of the endosperm.

To complete this study, we sought to document tannic cell walls in other parts of the seed coat further by comparing their occurrence in WT and *tt4* seed coats.

During seed maturation, differentiating ii1′ and ii2 layers form the bpl. TEM observations indicated that the bpl is essentially composed of cell walls, consistent with previous reports (Figure 2e,f) (Beeckman *et al*., 2000; Kitamura *et al*., 2004). Indeed, no (or very few) cytoplasmic remains were visible in the ii1′ and ii2 layers (Figures 2e,f and S2), and the bpl appeared to be formed by the close apposition of ii1′ and ii2 cell walls with the outer ii1 and the inner oi1 cell walls. This conclusion is further supported by CW staining, showing extensive labeling of cellulose throughout the bpl (Figure S4).

Furthermore, the TEM observations in mature seeds reported here show that ii1 cell walls and the bpl have a similar electron density so that their delimitation is sometimes difficult to discern, consistent with the notion that the bpl is also made of cell walls impregnated by tannins, as previously shown (Figures 2e,f and S2) (Beeckman *et al*., 2000; Kitamura *et al*., 2004; Pourcel *et al*., 2007). Consistent with this view, in *tt4* seeds the bpl had an electron‐lucent appearance in TEM similar to that of ii1 cell walls (Figure S9). In addition, in unstained *tt4* histological sections, the bpl lost its brown coloration (Figure S1b,c).

Moreover, a clear signal was observed in the bpl upon staining with the tannin‐specific dye DMACA (Figures 1c and S3). Indeed, DMACA stainings of semi‐thin sections showed a strong signal in the entire bpl layer, similar to that observed in the ii1 layer; furthermore, this signal was absent in *tt4* mutant seeds (Figures 1c and S3b). These observations show that tannins are not only present in the ii1 layer but also in the bpl.

In WT seed sections (unstained, or upon staining with toluidine blue or methylene blue), the bpl did not have exactly the same color as that of the interior of ii1 cells and ii1 cell walls (Figures 1a,b and S1a,c); however, all these structures were recognized by DMACA, attesting that tannins are part of their composition. The variability in color may be due to additional components present in the bpl, such as other types of flavonoids (e.g. flavonols).

Thus, our observations confirm that tannic cell walls are major building blocks of the bpl, consistent with previous reports (Beeckman *et al*., 2000; Kitamura *et al*., 2004; Pourcel *et al*., 2007). Therefore, they support the notion further that tannic cell walls account for a substantial part of the mature Arabidopsis seed coat.

## DISCUSSION

*tt* mutant seeds have low viability and dormancy due to a defective seed coat. We sought to understand the origin of the intriguing permeability defects observed in the endosperm‐associated cuticle of *tt* mutants (*tt4*, *tt5*, *ban*, *tt12* and *tt15*) reported by Loubéry *et al*., 2018. Indeed, *tt* mutants are deficient not in cuticular components synthesis, but rather in flavonoid synthesis. This led us to identify a tannic cell wall tightly associated with the endosperm‐associated cuticle; furthermore, this component remains attached to the cuticle at the surface of the endosperm during seed coat rupture where it contributes, together with the cuticle, to the permeability properties of the endosperm. Thus, we interpret the permeability defects of the endosperm in *tt* mutants reported by Loubéry *et al*., 2018 because of the absence of tannins in the cell wall that is present, together with a cuticle, at the surface of the endosperm. It is remarkable that although the cuticle arises from ii1 cells it remains tightly attached, together with the ii1 tannic cell wall, to the surface of the endosperm after seed coat rupture. This suggests the existence of a specific mechanism implementing this tight adhesion that remains to be investigated.

Furthermore, we confirm that tannic cell walls are the main constituents of the bpl and therefore are major structural components of the mature seed coat, consistent with previous reports (Beeckman *et al*., 2000; Kitamura *et al*., 2004; Pourcel *et al*., 2007).

In the mature seed, ii1 tannic cell walls together with the bpl tannic cell walls define a large and continuous electron‐dense tannin‐rich region in the seed coat (Figures S2 and S5). Two electron‐lucent and previously characterized impermeable barriers border this structure: on the inner side, the endosperm‐associated cuticle and on the outer side, a suberized cell wall of outer integument cells (Yadav *et al*., 2014; De Giorgi *et al*., 2015; Gou *et al*., 2017; Loubéry *et al*., 2018). The presence of these two barriers may explain why tannins synthesized in ii1 cells diffuse to the bpl during seed development, but not further. Furthermore, tannins shield living tissues from external damage, but they in turn could interfere with vital functions: hence, tannin containment within the seed coat may be crucial to avoid exposing living tissues to these potentially harmful compounds.

### Reinterpretation of the two‐layered structure observed in TEM at the outer surface of endosperm cells

In a previous report, the two‐layered structure observed in TEM at the outer surface of endosperm cells, consisting of an inner electron‐lucent layer and an outer electron‐dense layer was viewed as a cuticle made of two different compositions (De Giorgi *et al*., 2015; Loubéry *et al*., 2018).

In the scientific literature, cuticles are predominantly described as electron‐dense layers, according to their appearance in TEM, whether in seeds, stem or leaves (Beeckman *et al*., 2000; Andème Ondzighi *et al*., 2008; Yang *et al*., 2008; DeBolt *et al*., 2009; Voisin *et al*., 2009; Bowman, 2011; Shumborski *et al*., 2016; Moussu *et al*., 2017). However, previous reports have described amorphous electron‐dense procuticles that are converted to a simple electron‐lucent amorphous cuticle (Jeffree, 2006). Thus, the electron‐dense character of a layer observed in TEM is not an absolute criterion to define a cuticle and several cuticles have been described as partially or entirely electron‐lucent (Jeffree, 2006; Budke *et al*., 2011; Guzmán *et al*., 2014; Bourgault *et al*., 2020). Interestingly, examining the two‐layer structure that borders the endosperm throughout development suggests a picture that is consistent with a model of a cuticle progressively losing its electron‐dense character. Indeed, the endosperm‐associated two‐layered structure is of maternal origin, derived from ii1 cells and transferred to the endosperm between the torpedo and walking‐stick stage (Loubéry *et al*., 2018). During the earlier stages of seed development (globular and heart stages), the structure is present at the surface of ii1 cells and is made of two layers but with an inverted electron density relative to that found in mature seeds. Indeed, during the early stages, the cuticle is electron‐dense, whereas the ii1 cell wall is rather electron‐lucent (Loubéry *et al*., 2018). From the mature embryo stage onwards the cuticle is no longer electron‐dense; in contrast, the ii1 cell wall becomes electron‐dense only at late seed maturation stages.

Thus, we conclude that, in mature seeds, of the two layers at the outer surface of the endosperm only the inner layer is made of cuticular material, the outer layer corresponding to the tannin‐impregnated ii1 cell wall.

### Proposed functions of tannins in the seed coat

In Arabidopsis seeds, the absence of flavonoids correlates with higher seed permeability to water and oxygen; in turn, this lowers seed dormancy and viability (Debeaujon *et al*., 2000; Chahtane *et al*., 2017). Tannins can act as antioxidants and may thus limit oxygen diffusion in the seed coat (Harborne and Williams, 2000; Pietta, 2000; Debeaujon *et al*., 2007; Pourcel *et al*., 2007). Furthermore, some tannins are known to be impermeable to water (Debeaujon *et al*., 2007). The permeability experiments reported by Loubéry *et al*., 2018, showing that the *tt* mutant endosperm is more permeable to toluidine blue in seed coat‐ruptured seeds, together with the evidence presented here that the tannic cell wall remains associated with the endosperm upon seed coat rupture, provides further direct evidence that the tannic cell wall renders the seed coat less permeable to outer compounds.

In seeds deprived of tannins, such as *tt4* mutant seeds, the ii1 cell layer is considerably more collapsed than in WT seeds (Figure 1b,c), which is consistent with the thinner and weaker seed coats of *tt4* mutant seeds (Debeaujon *et al*., 2000). Interestingly, it is known that tannins interact with cell wall components, by making cross‐links with proteins and polysaccharides (Marles *et al*., 2003; Debeaujon *et al*., 2007), thus strengthening the whole structure. The presence of tannins in ii1 cell walls could then increase the mechanical stability of these cells and globally enhance the hardiness of the seed coat. Furthermore, it has been demonstrated that artificial incorporation of flavonoids in wood cell walls improves wood stability upon changes in temperature and humidity; it also enhances its durability by reducing the moisture content, hence decreasing the risk of degradation by fungi (Ermeydan *et al*., 2012). This is consistent with the notion that the presence of tannins in ii1 cell walls and the bpl improve Arabidopsis seed solidity and durability.

The list of flavonoids functions that have been proposed or demonstrated is extensive: flavonoids have been shown in particular to act as signaling molecules between plants and other organisms, delivering messages of attraction, repulsion or inhibition. Arabidopsis plant roots are known to maintain relations with microbial communities, which can positively affect their health and development (Bergelson *et al*., 2019; Cheng *et al*., 2019). As it is established that flavonoids can act as signaling molecules with soil organisms, we can speculate that tannins from the seed coat could prime the development of these symbiotic relations for the upcoming roots. Interestingly, we showed that seed coat rupture occurs at the level of anticlinal ii1 cell walls, which is expected to release the tannins they contain. Consistent with this notion, patches stained by DMACA are occasionally detectable on the endosperm surface upon seed coat rupture (brown arrowheads in Figures 3b and S10), but in most cases they do not appear to remain attached to the surface of the endosperm, suggesting that they are released in the seed environment. Thus, it is tempting to speculate that if tannins were to have a role in the establishment of mutualistic interactions with microorganisms, the mechanics of seed coat rupture would directly time the release of tannins with the moment when they are needed. In addition, upon imbibition, seeds can suffer sudden abiotic stresses, such as dehydration, an osmotic disequilibrium or variations of temperature, which leads to a germination arrest until conditions are favorable again (Lopez‐Molina *et al*., 2001, 2002). Germination arrest could then take place in seeds that already ruptured their seed coat. During this intermediate developmental stage the two‐layer structure made of a cuticle and a tannic cell wall, together with the release of the ii1 cell tannin contents, may maintain a certain level of impermeability to water and oxygen, as well as some resistance to pathogens and herbivores.

Another widespread role for flavonoids (including tannins) that has been described in various species is in the defense against herbivores or pathogenic bacteria and fungi. Tannin toxicity is due to their complexation with proteins or substrates, alteration of membranes or complexation with metal ions, which causes severe limitations for pathogen health and growth (Scalbert, 1991; Chung *et al*., 1998; Shirley, 1998; Dixon *et al*., 2005). Seeds are known to be the target of deleterious fungi and bacteria: indeed, seeds infected with fungi or bacteria deteriorate faster and have a lower viability than uninfected seeds (Mohamed‐Yasseen *et al*., 1994). One putative role of tannic walls in the seed could be to shield the seed against soil microorganisms so as to protect the embryo from pathogenic attacks. Concerning bigger predators, it has been shown that some animals avoid seeds with a high tannin content, which is probably due to the astringency of tannins, and consequently either avoid consuming seeds with a high tannin content or reject the seeds after fruit consumption (Harborne and Williams, 2000; Zhang *et al*., 2013; Ancillotto *et al*., 2015). This might be beneficial for the plant as well as for the consumer. Indeed, in both cases the seed is not destroyed by the animal and its dispersion is facilitated.

### Development and presence of different protective layers in the seed coat

In the Arabidopsis seed coat, several layers participate in the protection of the embryo and the endosperm. To face various sources of stress, these layers have different compositions: a cuticle along the endosperm surface; tannins inside ii1 cells and in their walls, as well as in the bpl; suberin and flavonols in oi1 cells (Pourcel *et al*., 2005; Yadav *et al*., 2014; Gou *et al*., 2017). We showed that from seed coat rupture onwards, the endosperm is covered by a two‐layered maternal structure made of a cuticle and a tannic cell wall, deriving from ii1 cells, and conferring a supplemental level of defense and protection. Both components appear to have similar functions, in particular for the control of water and oxygen passage, ensuring a high level of protection in this regard. In addition, tannins released by ii1 cells upon seed coat rupture may bring additional putative functions regarding the interactions of Arabidopsis seeds with pathogenic or beneficial organisms, which remain to be investigated.

## EXPERIMENTAL PROCEDURES

### Plant material and growth conditions

Arabidopsis (*Arabidopsis thaliana*) Columbia seeds were used as WT material together with the *tt4‐5* mutant in the Columbia background (NASC stock number N66121, contributed by F. Zhang, Y. Qi and D. Voytas). For each experiment presented, the seed material used (i.e. the WT seed material and the mutant seed material) was harvested on the same day from plants grown under identical environmental conditions. Dry siliques were obtained at about 8 weeks after planting. The number of seeds analyzed for each set of experiments and genotypes is presented in Table 1.

**Table 1:** Biological sample sizes

Semi‐thin sections			Whole‐mount preparations			TEM		SEM	
Toluidine blue	Col‐0	>40	Unstained	Col‐0	>40	Col‐0	10	Col‐0	>40
	*tt4*	>40	DMACA	Col‐0	>40	*tt4*	6		
Methylene blue	Col‐0	>40		*tt4*	>40				
	*tt4*	>40	Auramine O	Col‐0	>40				
Unstained	Col‐0	>60	CW	Col‐0	>40				
	*tt4*	>40							
DMACA	Col‐0	>60							
	*tt4*	>40							
Auramine O	Col‐0	>60							
	*tt4*	>40							
CW	Col‐0	>40							
	*tt4*	>40							

### Semi‐thin sections

Eight‐hour‐imbibed seeds were delicately punctured with a fine needle and fixed overnight at 4°C in 2.5% (v/v) glutaraldehyde and 0.01% (v/v) Tween‐20 in phosphate buffer (pH 7.2) after vacuum infiltration. Samples were then washed with water, embedded in pellets of 1.5% (w/v) agarose, dehydrated in a graded ethanol series and embedded in Epon 812. Finally, 1 μm semi‐thin sections were cut using a UCT microtome (Leica, Wetzlar, Germany) and placed on SuperFrost slides (Roth AG, Arlesheim, Switzerland). For DMACA stainings, seed sections were incubated in the dark during 72 h at room temperature in a solution of 0.3% (w/v) DMACA (Sigma‐Aldrich Chemie GmbH, Buchs, Switzerland) in a mixture of methanol and 6 m HCl (1:1, v/v). To prepare this solution the DMACA powder was first solubilized in methanol and agitated for 10 min, then H_2_O and HCl were added to the mixture. The slices were finally rinsed several times with 50% ethanol and mounted in water. For toluidine blue staining, fixed seed sections were incubated in a solution containing 0.5% (w/v) toluidine blue in 0.1 m phosphate buffer (pH 6.8) during 5 min at 70°C on a hot plate, rinsed with H_2_O and mounted in Eukitt. For methylene blue staining, a stock solution was prepared containing 0.15% (w/v) methylene blue in 10% glycerol, 10% methanol, 30% distilled water and 50% phosphate buffer (pH 6.9); the solution was then diluted 20× in distilled water and fixed seed sections were incubated during 5 min at 70°C on a hot plate, rinsed with H_2_O and mounted in Eukitt. For CW stainings, sections were first incubated for 2 min in 0.01% (w/v) CW in water, then washed with phosphate‐buffered saline (PBS; pH 6.8) and mounted in PBS (pH 6.8) with glycerol (1:1, v/v). In Figures 1a (center bottom) and S1b,c, non‐stained slices were simply mounted in Eukitt.

### Paraffin sections

Eight‐hour‐imbibed seeds were delicately punctured with a fine needle and fixed overnight at 4°C in phosphate buffer (pH 7.2) with 4% (v/v) formaldehyde and 0.25% (v/v) glutaraldehyde, after vacuum infiltration. Samples were then embedded in pellets of 1.5% (w/v) agarose, dehydrated in a graded ethanol series, cleared in Neoclear and embedded in paraffin. Sections (8 μm thick) were cut with a Cut 4050 microtome (microTec Laborgeräte GmbH, Walldorf, Germany), placed on SuperFrost slides (Roth AG), deparaffinized with Neoclear and rehydrated with water. For Auramine O staining, sections were incubated for 5 min in 0.001% (w/v) Auramine O in water, then washed with PBS (pH 6.8) and mounted in PBS (pH 6.8) with glycerol (1:1, v/v).

### Whole‐mount stainings

Fresh seeds were plated on Murashige and Skoog medium supplemented with 10 μm abscisic acid (ABA) during 36 h (until seed coat rupture occurred). For double CW/Auramine O stainings, seeds were first incubated for 20 min in 0.01% (w/v) CW in water, then washed with water and incubated for 30 min in 0.005% (w/v) Auramine O in water; finally, they were washed with water and mounted in water between the slide and coverslip. For DMACA stainings, seeds were incubated during 1 h in a DMACA solution (see above), rinsed several times with 50% ethanol and mounted in water between the slide and coverslip. In Figure 3a, non‐stained seeds were simply mounted in water between the slide and coverslip.

### Light microscopy

Samples were examined with an Eclipse 80i widefield microscope (Nikon, Tokyo, Japan) equipped with a 40× Plan Fluor numerical aperture (NA) 0.75 air lens and a Digital Sight DS‐Fi1 color CCD camera (Nikon) or a DS‐Fi3 CMOS color camera (Nikon). Fluorescence excitation was done with an Intensilight C‐HGFI mercury vapor lamp (Nikon); CW was examined using a 4′,6‐diamino‐phenylindole filter set (excitation, 352–402 nm; emission, 417–477 nm), Auramine O was examined using a cyan fluorescence protein filter set (excitation, 426–450 nm; emission, 467–499 nm) and for Figure 1a, autofluorescence was examined using a green fluorescence protein long‐pass filter (excitation, 460–500 nm; emission >510 nm). For Figure 2d, Auramine O was examined using an SP5 confocal microscope (Leica) equipped with a 63× PlanApo NA 1.4 oil lens, an argon laser with excitation at 458 nm, a HyD detector with emission collection between 483 and 513 nm, and using a pixel size of 100 nm; a maximal projection of a few z planes was performed and displayed. Whole‐mount samples in Figures 3b and S10 were examined with an Axio Zoom.V16 stereomicroscope (Zeiss) equipped with a 1× NA 0.25 objective and an Axiocam 512 CMOS camera, under episcopic illumination.

## TEM

TEM was performed as described previously (Loubéry *et al*., 2018). Briefly, 8‐h‐imbibed seeds were delicately punctured with a fine needle and fixed overnight at 4°C in 2.5% (v/v) glutaraldehyde and 0.01% (v/v) Tween‐20 in 100 mm sodium cacodylate (pH 7) after vacuum infiltration. After a primary post‐fixation in 1.5% (v/v) osmium tetroxide for 2 h at 4°C and a secondary post‐fixation in 1% (w/v) uranyl acetate for 1 h at 4°C, seeds were embedded in pellets of 1.5% (w/v) agarose, dehydrated in a graded ethanol series and embedded in Epon 812. Then, 85 nm ultra‐thin sections were cut using a UCT microtome (Leica), stained with 2.5% (w/v) uranyl acetate and Reynolds lead citrate, and finally observed with a Tecnai G2 Sphera (ThermoFischer Scientific, Hillsboro, OR, USA) at 120 kV, equipped with a high‐resolution digital camera.

## SEM

Fresh seeds were plated on Murashige and Skoog medium supplemented with 10 μm ABA during 36 h (until seed coat rupture occurred). Seeds were delicately transferred on to SEM holders and placed on double‐sided carbon tape (Electron Microscopy Sciences, Hatfield, PA, USA), and afterwards treated with a gold sputter coater (JFC‐1200; JEOL, Tokyo, Japan). Imaging was performed with a JSM‐6510LV (JEOL) SEM in high vacuum mode with a spot size of 40, an acceleration of 15 kV and a working distance of 12 mm.

### Image treatment and analysis

Images were treated and analyzed using the software Fiji (Schindelin *et al*., 2012). For both histology and TEM, tiling and stitching were used to obtain high‐resolution large fields of view. Tiling was performed manually, and stitching was done using the Fiji MosaicJ plugin (Thévenaz and Unser, 2007). The left panel in Figure 1a was modified using Adobe Photoshop Lightroom CC version 2015.12 and Adobe Photoshop CC 2017. In Figures 3c and S3a, CW stainings were colorized with the Fiji Magenta and Grays look‐up tables, respectively; in Figure 2d, Auramine O staining was colorized with the Fiji Fire look‐up table, to provide a larger dynamic range and a higher quality of visualization. All figures were mounted using Adobe Illustrator CC 22.0.0.

## AUTHOR CONTRIBUTIONS

LD, SL and LL‐M conceived the experiments; LD, AU‐P and SL performed the experiments; LD, SL and LL‐M analyzed the data; LD drew the artwork; LD, SL and LL‐M wrote the article; LL‐M secured the funding for the project.

## CONFLICTS OF INTEREST

The authors declare that they have no competing interests.

## Supporting information


**Figure S1**. Presence of tannins at the outer surface of the endosperm.
**Figure S2**. Presence of an extended electron‐dense reticulated structure surrounding the endosperm.
**Figure S3**. Examples of seed coat sections stained with DMACA.
**Figure S4**. Seed coat section stained with CW.
**Figure S5**. Tannic cell walls and the endosperm‐associated cuticle.
**Figure S6**. Size and morphology of embryonic, endosperm and ii1 cells in seed coat‐ruptured seeds.
**Figure S7**. SEM reveals the pattern of ii1 cells at the surface of the endosperm upon seed coat rupture.
**Figure S8**. SEM reveals endosperm cells beneath the pattern of ii1 cells left at the surface of the endosperm.
**Figure S9**. The inner seed coat and the endosperm surface region in *tt4* seeds.
**Figure S10**. Presence of tannins at the surface of the endosperm upon seed coat rupture.

## Data Availability

All relevant data can be found within the manuscript and its supporting materials.
