# Supplementary material for: Identification of tannic cell walls at the outer surface of the endosperm upon Arabidopsis seed coat rupture
Source: Plant J. 2020 Oct 15;104(3):567–80. doi: 10.1111/tpj.14994 (PMC7702108; doi:10.1111/tpj.14994)
Supplement: Supplementary file 1 — Figure S1. Presence of tannins at the outer surface of the endosperm. Figure S2. Presence of an extended electron‐dense reticulated structure surrounding the endosperm. Figure S3. Examples of seed coat sections stained with DMACA. Figure S4. Seed coat section stained with CW. Figure S5. Tannic cell walls and the endosperm‐associated cuticle. Figure S6. Size and morphology of embryonic, endosperm and ii1 cells in seed coat‐ruptured seeds. Figure S7. SEM reveals the pattern of ii1 cells at the surface of the endosperm upon seed coat rupture. Figure S8. SEM reveals endosperm cells beneath the pattern of ii1 cells left at the surface of the endosperm. Figure S9. The inner seed coat and the endosperm surface region in tt4 seeds. Figure S10. Presence of tannins at the surface of the endosperm upon seed coat rupture. [file TPJ-104-567-s001.pdf]

Supporting information for

Identification of tannic cell walls at the outer surface of the endosperm upon Arabidopsis seed coat rupture

Lara Demonsais[1], Anne Utz-Pugin[1], Sylvain Loubéry[1,*] and Luis Lopez-Molina[1,2,*]

[1]Department of Botany and Plant Biology, University of Geneva, Geneva, Switzerland
[2]Institute of Genetics and Genomics in Geneva (iGE3), University of Geneva, Geneva, Switzerland
[*] Co-corresponding authors

Corresponding authors:
Luis Lopez-Molina (Luis.LopezMolina@unige.ch)
Sylvain Loubéry (sylvain.loubery@unige.ch)

ORCID IDs: 0000-0001-6524-2850 (L.D.) 0000-0001-7061-9456 (S.L.); 0000-0003-0463-1187 (L.L-M.)

The following content includes:

Figure S1. Presence of tannins at the outer surface of the endosperm.

Figure S2. Presence of an extended electron-dense reticulated structure surrounding the endosperm.

Figure S3. Examples of seed coat sections stained with DMACA.

Figure S4. Seed coat section stained with CW.

Figure S5. Tannic cell walls and the endosperm-associated cuticle.

Figure S6. Size and morphology of embryonic, endosperm and ii1 cells in seed coat-ruptured seeds.

Figure S7. SEM reveals the pattern of ii1 cells at the surface of the endosperm upon seed coat rupture.

Figure S8. SEM reveals endosperm cells beneath the pattern of ii1 cells left at the surface of the endosperm.

Figure S9. The inner seed coat and the endosperm surface region in *tt4* seeds.

Figure S10. Presence of tannins at the surface of the endosperm upon seed coat rupture.

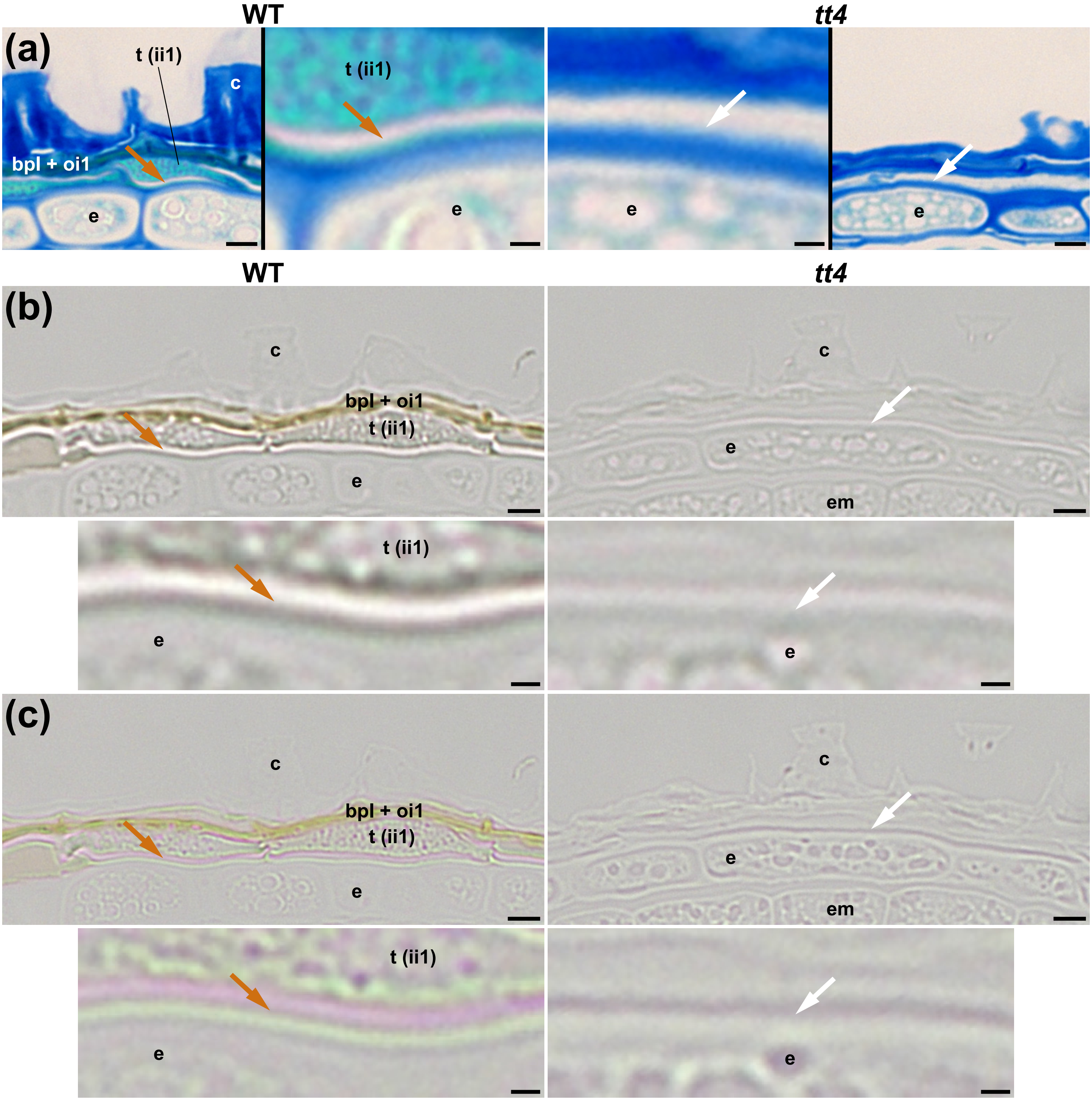

Figure S1. Presence of tannins at the outer surface of the endosperm.
Semi-thin sections of WT seeds (left panels) and *tt4* seeds (right panels) stained with methylene blue (a) or unstained (b,c). A global view of the seed coat and an enlargement of the region around the arrow is shown. (b,c) represent the same section observed on two different focal planes. Brown arrows indicate the detection of tannins along the endosperm outer cell wall; white arrows indicate no detection of tannins along this cell wall.
Scale bars = 5 µm and 1 µm for general views and magnified insets, respectively.
Abbreviations: bpl, brown pigment layer; c, columella; e, endosperm; em, embryo; ii1, inner integument 1; oi1, outer integument 1; t, tannin blocks.

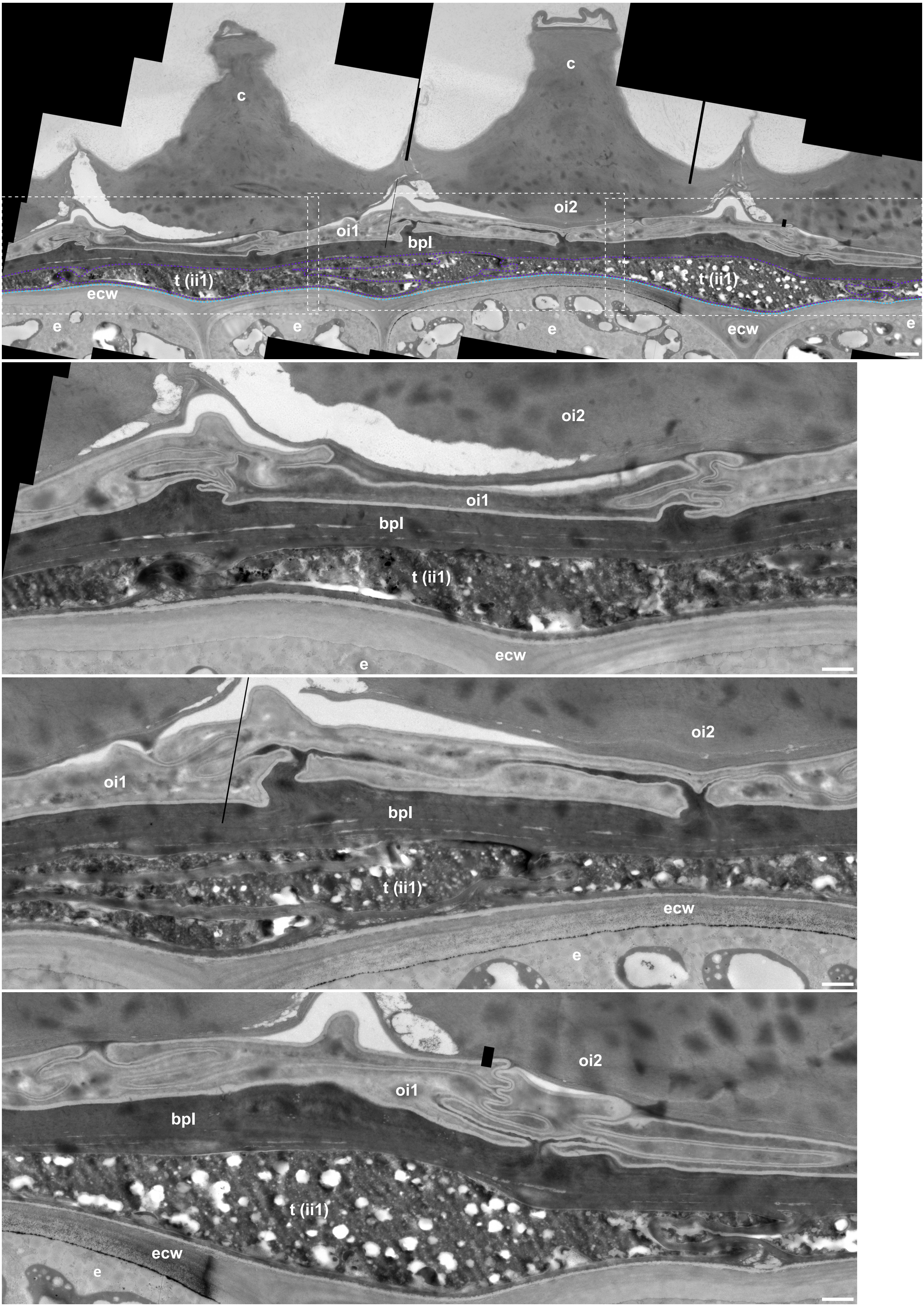
c
c
oi1
oi2
bpl
t (ii1)
t (ii1)
ecw
ecw
e
e
e
e
oi2
oi1
bpl
t (ii1)
ecw
e
oi2
oi1
bpl
t (ii1)
ecw
e
oi2
oi1
bpl
t (ii1)
ecw
e

Figure S2

Figure S2. Presence of an extended electron-dense reticulated structure surrounding the endosperm. High-resolution TEM view of a WT seed coat, adapted from Loubéry *et al.*, 2018. Dashed rectangles in the top image delineate the regions that are magnified in the three bottom panels. The purple dashed line indicates the cell walls of ii1 cells (electron-dense material) and the light blue dashed line indicates the endosperm-associated cuticle (electron-lucent material).

Scale bars = 2 µm and 1 µm for the general view and magnified insets, respectively.

Abbreviations: bpl, brown pigment layer; c, columella; e, endosperm; ecw, endosperm cell wall; ii1, inner integument 1; oi1, outer integument 1; oi2, outer integument 2; t, tannin blocks.

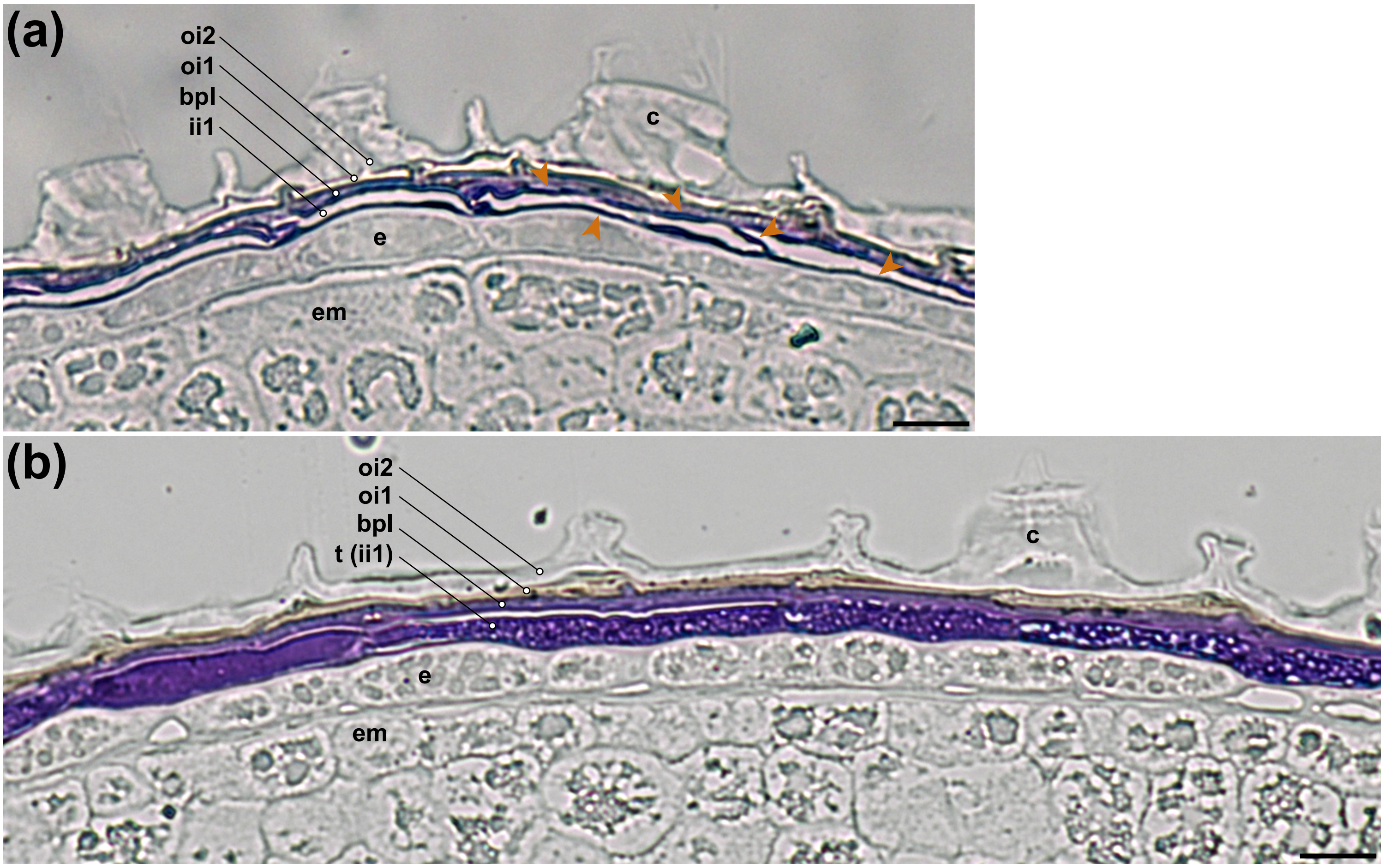

Figure S3. Examples of seed coat sections stained with DMACA.
Semi-thin sections of WT seeds stained with DMACA. Brown arrowheads in (a) indicate a portion of the reticulated structure formed by the ii1 tannic cell walls.
Scale bars = 10 µm.
Abbreviations: bpl, brown pigment layer; c, columella; e, endosperm; em, embryo; ii1, inner integument 1; oi1, outer integument 1; oi2, outer integument 2; t, tannin blocks.

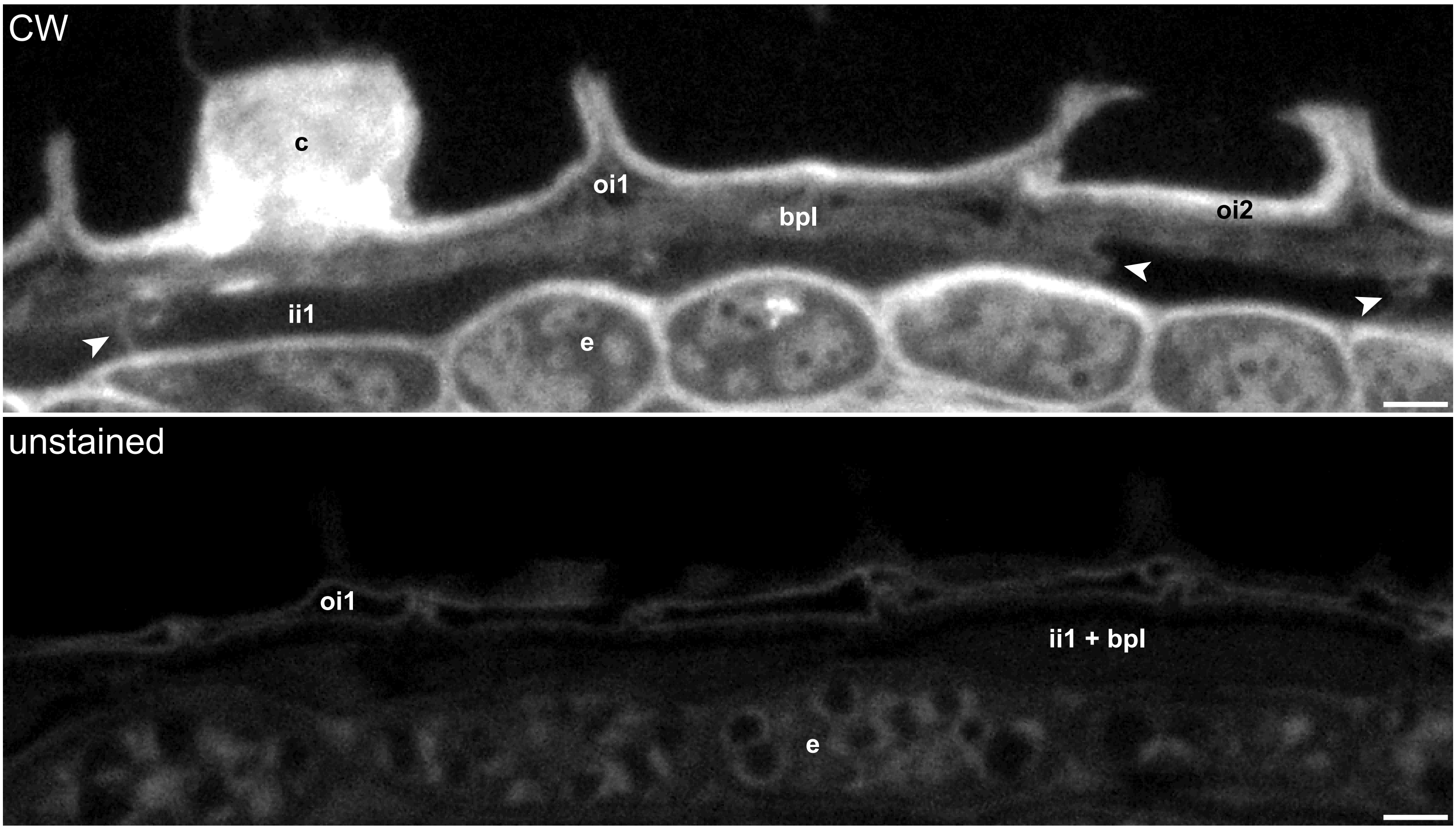

Figure S4. Seed coat section stained with CW.

Semi-thin sections of WT seeds stained with CW or unstained. White arrowheads indicate cellulose-containing anticlinal ii1 cell walls.

Scale bars = 10 µm.

Abbreviations: bpl, brown pigment layer; c, columella; e, endosperm; ii1, inner integument 1; oi1, outer integument 1; oi2, outer integument 2.

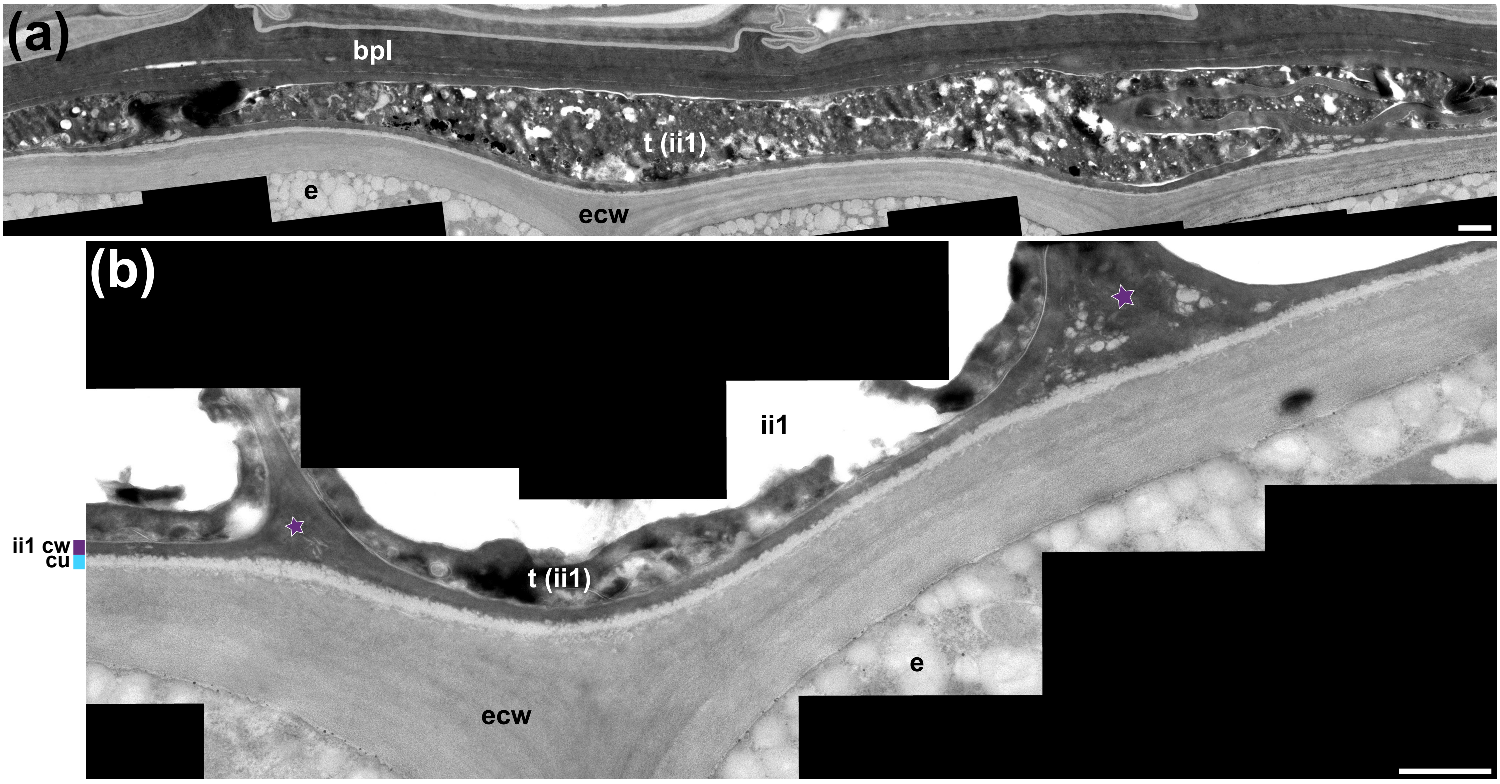

Figure S5. Tannic cell walls and the endosperm-associated cuticle.

(a,b) TEM micrographs of WT seed coat sections.

(a) Reproduction of the image depicted in Figure 2f, without the dashed lines. ii1 tannic cell walls together with the bpl tannic cell walls define a large and continuous electron-dense tannin-rich region in the seed coat.

(b) Electron-lucent droplet-like structures are not always clearly visible. Purple stars indicate triangles at the meeting points of adjacent ii1 cells and the cuticle which lack or are not extensively filled with electron-lucent droplet-like structures. The cyan mark indicates the endosperm-associated cuticle and the purple mark indicates the ii1 cell wall.

Scale bars = 1 µm.

Abbreviations: bpl, brown pigment layer; cu, cuticle; cw, cell wall; e, endosperm; ecw, endosperm cell wall; ii1, inner integument 1; t, tannin blocks.

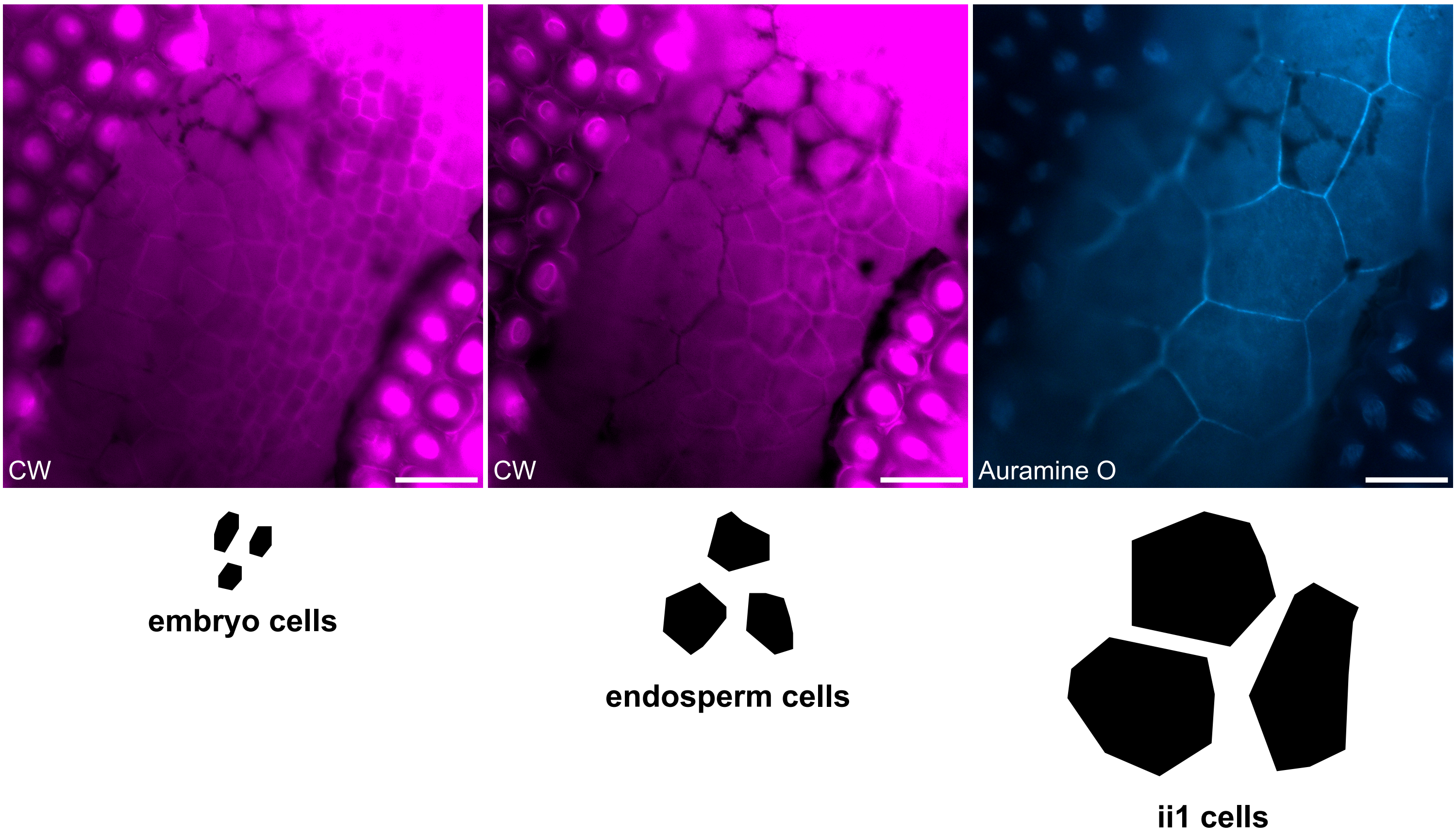

Figure S6. Size and morphology of embryonic, endosperm and ii1 cells in seed coat-ruptured seeds. Seed coat-ruptured WT seed stained with CW (left and center panels) and Auramine O (right panel). The three pictures represent the same seed observed on different focal planes in order to visualize the different cell layers. Black shapes under each picture show the shape and dimensions of a few cells in each layer, illustrating their differences in morphology and in size.
Scale bars = 50 µm.
Abbreviation: ii1, inner integument 1.

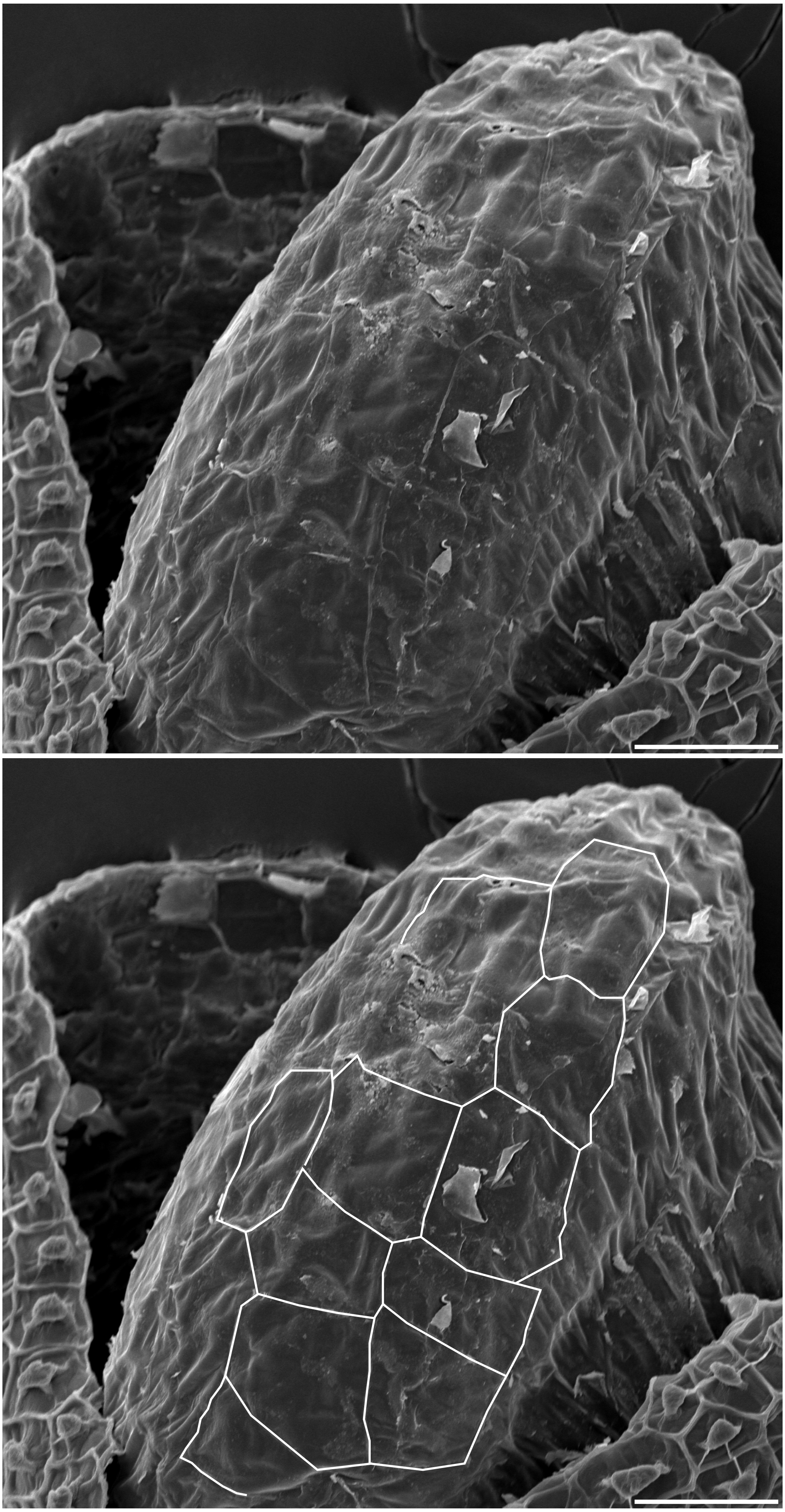

Figure S7. SEM reveals the pattern of ii1 cells at the surface of the endosperm upon seed coat rupture. SEM micrographs of the seed coat-ruptured WT seed depicted in Figure 3D. The top picture is without any annotation and in the bottom picture, white lines delineate the contours of ii1 cells.
Scale bars = 50 µm.

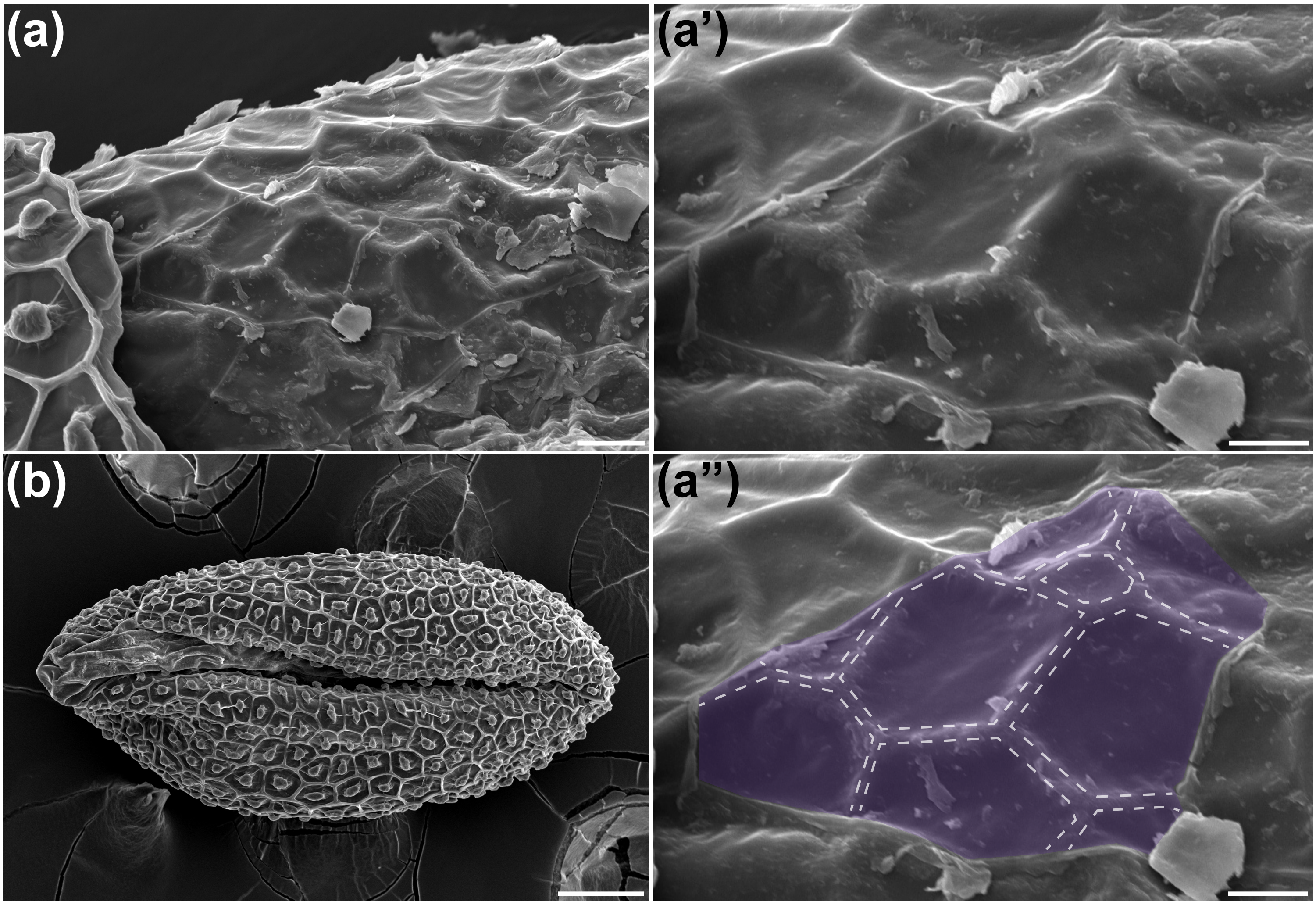

Figure S8. SEM reveals endosperm cells beneath the pattern of ii1 cells left at the surface of the endosperm.

(a, a' and a'') SEM micrograph of a seed coat-ruptured WT seed. In this region the seed surface was affected by the SEM vacuum: the cytoplasms of underlying endosperm cells collapsed slightly, giving them a hollow appearance and making their cell walls visible. (a') and (a'') are enlargements of the boxed region in (a). In (a''), the surface of one ii1 cell was colorized in purple, and white dashed contours highlight endosperm cell walls.

(b) Example of a seed coat-ruptured seed that closed itself as a result of the vacuum in the SEM, hiding the zones of interest (compare with Figures 3a,b or S10 for an image of a typical seed coat-ruptured seed without applied vacuum).

Scale bars = a, 20 µm; a' and a'', 10 µm; b, 100 µm.

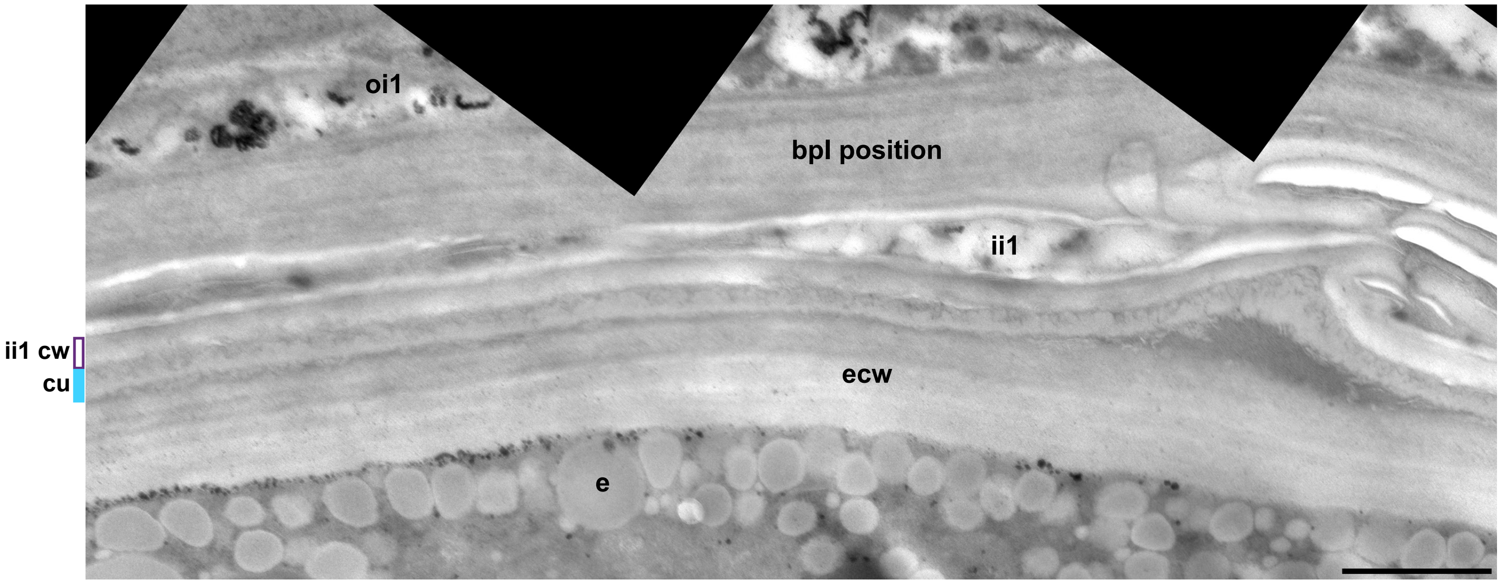

Figure S9. The inner seed coat and the endosperm surface region in *tt4* seeds.
TEM micrograph of a *tt4* seed coat. The region corresponding to the bpl position and ii1 cell walls is made of electron-lucent material. The cyan mark indicates the position of the cuticle and the purple mark indicates the position of the ii1 cell wall.
Scale bars = 1 µm.
Abbreviations: bpl, brown pigment layer; cu, cuticle; cw, cell wall; e, endosperm; ecw, endosperm cell wall; ii1, inner integument 1; oi1, outer integument 1.

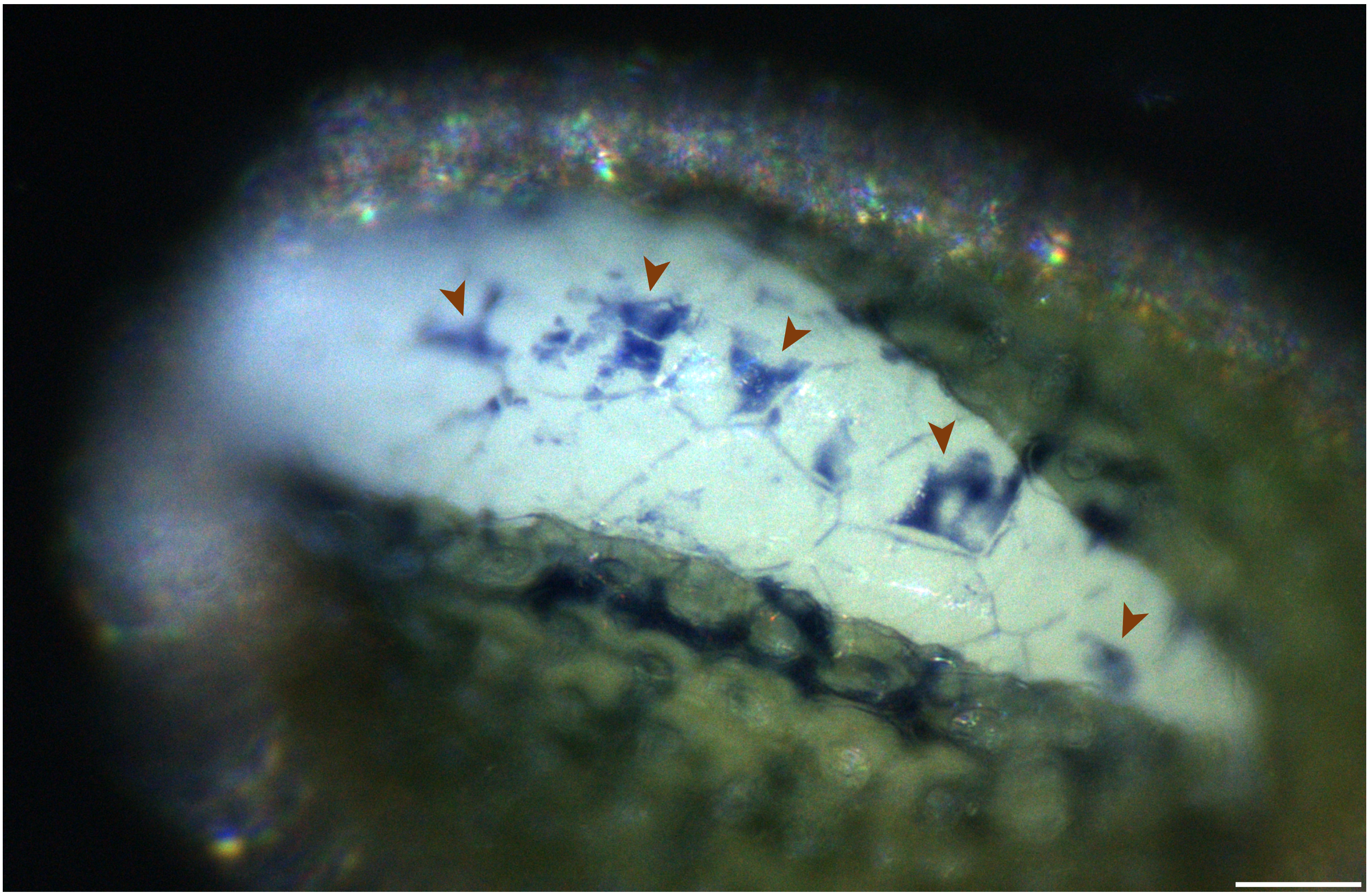

Figure S10. Presence of tannins at the surface of the endosperm upon seed coat rupture. A seed-coat ruptured WT seed was stained with DMACA. Brown arrowheads indicate patches of remaining tannin blocks (ii1).
Scale bar = 50 µm.
